# Hypothermic total liquid ventilation after experimental aspiration-associated acute respiratory distress syndrome

**DOI:** 10.1186/s13613-018-0404-8

**Published:** 2018-05-02

**Authors:** Jérôme Rambaud, Fanny Lidouren, Michaël Sage, Matthias Kohlhauer, Mathieu Nadeau, Étienne Fortin-Pellerin, Philippe Micheau, Luca Zilberstein, Nicolas Mongardon, Jean-Damien Ricard, Megumi Terada, Patrick Bruneval, Alain Berdeaux, Bijan Ghaleh, Hervé Walti, Renaud Tissier

**Affiliations:** 1 U955 – IMRB, Inserm, UPEC, Ecole Nationale Vétérinaire d’Alfort, 7 avenue du Général de Gaulle, 94700 Maisons-Alfort, France; 2 Paediatric and Neonatal Intensive Care Unit, Armand-Trousseau Hospital, UPMC, APHP, Paris, France; 3 Université de Sherbrooke, Sherbrooke, QC Canada; 4 Service d’Anesthésie et des Réanimations Chirurgicales, DHU A-TVB, Hôpitaux Universitaires Henri Mondor, Assistance Publique des Hôpitaux de Paris, Créteil, France; 5 UMR 1137, Inserm, Université Paris Diderot, Hôpital Louis Mourier, Réanimation Médico-chirurgicale, APHP, Colombes, France; 6 UMR 970, Inserm, Paris Cardiovascular Research Center, Hôpital Européen Georges Pompidou, Paris, France

**Keywords:** ARDS, Pneumonia, Aspiration, Total liquid ventilation, Hypothermia, Cardiac arrest

## Abstract

**Background:**

Ultrafast cooling by total liquid ventilation (TLV) provides potent cardio- and neuroprotection after experimental cardiac arrest. However, this was evaluated in animals with no initial lung injury, whereas out-of-hospital cardiac arrest is frequently associated with early-onset pneumonia, which may lead to acute respiratory distress syndrome (ARDS). Here, our objective was to determine whether hypothermic TLV could be safe or even beneficial in an aspiration-associated ARDS animal model.

**Methods:**

ARDS was induced in anesthetized rabbits through a two-hits model including the intra-tracheal administration of a pH = 1 solution mimicking gastric content and subsequent gaseous non-protective ventilation during 90 min (tidal volume [Vt] = 10 ml/kg with positive end-expiration pressure [PEEP] = 0 cmH_2_O). After this initial period, animals either received lung protective gas ventilation (LPV; Vt = 8 ml/kg and PEEP = 5 cmH_2_O) under normothermic conditions, or hypothermic TLV (TLV; Vt = 8 ml/kg and end-expiratory volume = 15 ml/kg). Both strategies were applied for 120 min with a continuous monitoring of respiratory and cardiovascular parameters. Animals were then euthanized for pulmonary histological analyses.

**Results:**

Eight rabbits were included in each group. Before randomization, all animals elicited ARDS with arterial oxygen partial pressure over inhaled oxygen fraction ratios (PaO_2_/FiO_2_) below 100 mmHg, as well as decreased lung compliance. After randomization, body temperature rapidly decreased in TLV versus LPV group (32.6 ± 0.6 vs. 38.2 ± 0.4 °C after 15 min). Static lung compliance and gas exchanges were not significantly different in the TLV versus LPV group (PaO_2_/FiO_2_ = 62 ± 4 vs. 52 ± 8 mmHg at the end of the procedure, respectively). Mean arterial pressure and arterial bicarbonates levels were significantly higher in TLV versus LPV. Histological analysis also showed significantly lower inflammation in TLV versus LPV group (median histological score = 3 vs. 4.5/5, respectively; *p* = 0.03).

**Conclusion:**

Hypothermic TLV can be safely induced in rabbits during aspiration-associated ARDS. It modified neither gas exchanges nor respiratory mechanics but reduced lung inflammation and hemodynamic failure in comparison with LPV. Since hypothermic TLV was previously shown to provide neuro- and cardio protective effects after cardiac arrest, these findings suggest a possible use of TLV in the settings of cardiac arrest-associated ARDS.

## Background

After out-of-hospital cardiac arrest and successful resuscitation, a post-cardiac arrest syndrome occurs and makes patients prone to infectious complications [1, 2]. Among them, early-onset pneumonia affects up to two-thirds of successfully resuscitated patients and are mainly related to aspiration. In this setting, aspiration pneumonia deteriorates pulmonary function and has negative impact on outcome [1–3]. More dramatically, aspiration pneumonia can culminate into acute respiratory distress syndrome (ARDS). For instance, around six percent of the patients resuscitated after out-of-hospital cardiac arrest present ARDS [4–6].

Hypothermic total liquid ventilation (TLV) could be an interesting alternative to conventional ventilation in the context of cardiac arrest-associated aspiration pneumonia since this technique has been previously shown to limit the post-cardiac arrest syndrome in animal studies [7–11]. During TLV, the lungs can be alternatively filled and emptied by perfluorocarbons, authorizing lung lavage as well as gas and thermal exchanges. This approach provides ultrafast cooling [12] and powerful neuro- and cardioprotection after cardiac arrest, as well as attenuation of multi-organ dysfunction after low perfusion states [7–11].

In the context of aspiration-associated ARDS, hypothermic TLV might also provide benefits per se through lung lavage or anti-inflammatory effects of liquid ventilation [13–18]. Conversely, one would also argue that TLV could increase the risk of ventilation-induced injury when initiated after such lung injury. Our previous reports with hypothermic TLV were indeed done in animal models of cardiac with very controlled conditions preventing accidental aspiration. Accordingly, the goal of the present study was to investigate whether TLV could be safely induced in a model of aspiration-associated ARDS and whether it modifies gas exchanges, respiratory mechanics, hemodynamic status or pulmonary inflammatory response in rabbits.

## Methods

The animal instrumentation and the ensuing experiments were approved by the institutional review board for animal research (project 04585-04 evaluated by the “Ethical committee Anses-Enva-UPEC”).

### Animal instrumentation

Male New Zealand rabbits (2.5–3.5 kg) were anesthetized using zolazepam, tiletamine and pentobarbital (all 20–30 mg/kg i.v.). After intubation, they were artificially ventilated with inspired fraction of oxygen (FiO_2_) of 30% (SAR-830P, CWE Inc., Ardmore, USA). Tidal volume was set at 8 mL/kg and respiratory rate at 30 cycles/min. Positive end-expiratory pressure (PEEP) was set at 5 cmH_2_O. Peripheral catheters were inserted into the ear marginal vein and artery for blood sampling and pressure monitoring, respectively. Temperature probes were also inserted into the rectum and esophagus. Anesthesia was maintained with additional administration of pentobarbital (5 mg/kg/h i.v.).

### Experimental protocol

After stabilization, rabbits were paralyzed by vecuronium bromide (0.4 mg/kg/h, i.v.). ARDS was induced with a two-hits injury including an intra-tracheal administration of 4 ml/kg of an aqueous solution at pH = 1 and a period of conventional non-protective gas ventilation. The acid solution was administered slowly into the tracheal tube, while animals were manipulated in order to improve lung distribution. Conventional non-protective ventilation consisted in mechanical ventilation with tidal volumes set at 10 ml/kg and PEEP at 0 cmH_2_O. Inhaled fraction of oxygen (FiO_2_) was increased to 100%. Conventional non-protective ventilation was maintained during 90 min according to preliminary experiments demonstrating an optimal balance between the occurrence of reproducible ARDS and the need for a sufficient survival and follow-up after group allocation. Body temperature was maintained around 38.5 °C throughout ARDS induction phase.

**Fig. 1:**
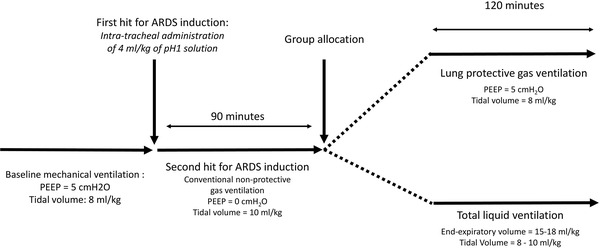
Schematic representation of the experimental protocol. *ARDS acute respiratory distress syndrome; PEEP positive end*-*expiratory pressure*

After the period of conventional ventilation, animals were randomly allocated to a group treated by lung protective gas ventilation (LPV) or TLV for 120 min (Fig. 1). In the LPV group, tidal volume was set at 8 ml/kg with PEEP = 5 cmH_2_O. Such tidal volumes used are slightly higher that the recommended volumes for humans with ARDS. However, lower tidal volumes are currently recommended in rabbits. Body temperature was maintained at 38.5 °C using heating pads in the LPV group. In the TLV group, animals were ventilated with a dedicated prototype of liquid ventilator continuously controlling liquid pressures and volumes (Inolivent-5, Université de Sherbrooke, QC, Canada) [16, 19]. They were submitted to hypothermia with a target temperature of 33 °C [7–11, 20, 21]. Lungs were initially filled with 13 ml/kg of perfluoro-n-octane (PFO, C8F18; F2-chemical^®^, Preston, Lancashire, UK). Tidal volume was set at 8-10 ml/kg and respiratory frequency at 9 cycles/min. The pulmonary end-expiratory volumes of PFO were increased to reach 15–20 ml/kg. The temperature of the liquid was initially set at 20 °C and progressively increased in order to maintain body core temperature at 33 °C. Static lung compliance was calculated by dividing tidal volume by the difference between airways pressures between end-inspiratory and end-expiratory pauses. Pause pressures were calculated after valve closures at the end of inspiration and expiration, respectively. Mean values were obtained with at least 3 cycles. After 120 min of LPV or TLV, animals were weaned back to conventional gas ventilation. Both groups were submitted to broncho-alveolar lavage at the end of the protocol for the further evaluation of albumin content. Immediately after broncho-alveolar lavage, animals were then euthanized using a lethal dose of pentobarbital (60 mg/kg iv). Lungs were collected, perfused and fixed with formaldehyde (4%) for histological analysis.

### Investigational parameters

Throughout the procedure, heart rate and arterial blood pressure were continuously monitored (HEM version 4.2, Notocord, Croissy-sur-Seine, France), as well as esophagus and rectal temperatures. Arterial blood samples were withdrawn at baseline, after ARDS induction and 30 and 120 min after the onset of LPV or TLV for the evaluation of arterial pH, bicarbonates blood levels, partial pressures of oxygen (PaO_2_) and carbon dioxide (PaCO_2_). Blood levels of interleukin 1β were also evaluated (ELISA Kit for interleukin 1 beta, SEA563Rb, Cloud-Clone, Katy, TX, USA). Albumin concentration was evaluated in the broncho-alveolar lavage solution.

In addition, lungs were prepared for histological analysis, as previously described [9]. The severity of ARDS lesions was assessed by a pathologist blinded to the experimental group. Two separate scores were attributed to each lung after the evaluation of all pulmonary lobes, i.e., one score for the magnitude of lung congestion and edema (0 = normal histological appearance; 5 = extensive congestion and edema in the entire lung) and one score for inflammatory lesions (0 = normal histological appearance; 5 = extensive leukocytic alveolitis with haline membranes), respectively.

### Statistical analysis

Data were expressed as mean ± SEM. Hemodynamic, respiratory and biochemical variables were compared between groups using a two-way ANOVA for repeated measures followed by a post hoc Holm–Sidak test if necessary. In order to reduce the number of comparisons, post hoc comparisons were performed between groups for each time point but not between time points within the same group. Histological scores were compared between groups using a Mann–Whitney *U* test. Significant differences were determined at *p* value ≤ 0.05.

## Results

### Baseline characteristics and ARDS induction

Eight rabbits were included in each LPV and TLV groups. As shown in Tables 1 and 2, no difference was observed at baseline among groups regarding body weight (3.1 ± 0.1 vs. 3.1 ± 0.1 kg, respectively), body temperature, hemodynamic or blood biochemical parameters. At the end of the conventional non-protective ventilation period, typical signs of ARDS were observed in both groups, including decreased lung compliance and decreased PaO_2_/FiO_2_ ratios with no difference between groups. The latter ratio achieved 41 ± 5 and 51 ± 9 mmHg at the end of the conventional ventilation period in LPV and TLV groups, as compared to 755 ± 97 and 730 ± 150 mmHg at baseline, respectively. FiO_2_ was maintained at 100% in all animals after ARDS induction.

**Table 1:** Hemodynamic parameters and temperatures throughout protocol in rabbits presenting experimental acute respiratory distress syndrome and treated by gaseous lung protective ventilation (LPV) or total liquid ventilation (TLV), respectively

Parameters and groups	Baseline	Conventional ventilation		LPV or TLV		
		15 min	90 min	10 min	60 min	120 min
*Esophageal temperature* (°C)						
LPV	38.7 ± 0.5	38.1 ± 0.6	38.2 ± 0.4	38.2 ± 0.4	38.6 ± 0.4	38.6 ± 0.4
TLV	38.5 ± 0.8	38.0 ± 0.8	38.8 ± 0.5	32.6 ± 0.6*	33.1 ± 0.3*	33.2 ± 0.2*
*Rectal temperature* (°C)						
LPV	38.7 ± 0.5	38.1 ± 0.6	38.2 ± 0.4	38.3 ± 0.3	38.4 ± 0.3	38.4 ± 0.4
TLV	38.7 ± 0.5	37.8 ± 0.9	39.1 ± 0.4	34.8 ± 0.5*	33.0 ± 0.2*	33.0 ± 0.1*
*Heart rate* (beats/min)						
LPV	257 ± 18	233 ± 16	246 ± 17	255 ± 17	248 ± 15	253 ± 7
TLV	242 ± 17	242 ± 17	262 ± 15	171 ± 6*	172 ± 6*	165 ± 7*
*Mean arterial blood pressure* (mmHg)						
LPV	75 ± 7	74 ± 6	65 ± 6	67 ± 5	61 ± 4	56 ± 8
TLV	69 ± 5	73 ± 3	65 ± 5	69 ± 6	82 ± 6*	74 ± 9*
*Lung compliance* (ml/kg/cmH_2_O)						
LPV	0.99 ± 0.11	0.59 ± 0.10	0.56 ± 0.09	0.78 ± 0.16	0.78 ± 0.16	0.65 ± 0.10
TLV	1.09 ± 0.18	0.61 ± 0.07	0.63 ± 0.09	0.74 ± 0.09	0.71 ± 0.07	0.67 ± 0.06

Statistical comparisons were only made for group effect but not among time points

*n *= 8 *in each LPV and TLV group*

*p < 0.05 versus LPV

**Table 2:** Biochemical characteristics throughout protocol in rabbits presenting experimental acute respiratory distress syndrome and treated by gaseous lung protective ventilation (LPV) or total liquid ventilation (TLV), respectively

Parameters and groups	Baseline	Conventional ventilation (*t* = 90 min)	LPV or TLV	
			30 min	120 min
*Arterial blood saturation* (%)				
LPV	100 ± 0	76 ± 4	62 ± 9	80 ± 5
TLV	100 ± 0	73 ± 3	83 ± 12	90 ± 2
*Arterial blood pH*				
LPV	7.36 ± 0.03	7.15 ± 0.06	7.14 ± 0.15	7.18 ± 0.05
TLV	7.35 ± 0.04	7.22 ± 0.02	7.17 ± 0.04	7.16 ± 0.04
*Arterial blood PaO*_*2*_ (mmHg)				
LPV	227 ± 29	41 ± 5	62 ± 9	52 ± 8
TLV	219 ± 38	51 ± 9	83 ± 12	62 ± 4
*Arterial blood PaO*_*2*_*/FiO*_*2*_ (mmHg)				
LPV	755 ± 97	41 ± 5	62 ± 9	52 ± 8
TLV	730 ± 150	51 ± 9	83 ± 12	62 ± 4
*Arterial blood PaCO*_*2*_ (mmHg)				
LPV	47 ± 3	70 ± 11	68 ± 7	67 ± 7
TLV	49 ± 6	72 ± 4	80 ± 5	79 ± 4
*Bicarbonate blood level* (mmol/l)				
LPV	28 ± 2	24 ± 2	23 ± 1	24 ± 2
TLV	28 ± 1	29 ± 2	30 ± 1*	31 ± 2*

Statistical comparisons were only made for group effect but not among time points

*n* = 8 *in each LPV and TLV group; Fi02, inhaled fraction of oxygen*

*p < 0.05 versus LPV

### Effect of TLV on gas exchanges, airways pressures, lung volumes and compliance

As shown in Table 2, blood oxygen saturation, pH, PaO_2_ and PaCO_2_ were not significantly different during TLV versus LPV. The PaO_2_/FiO_2_ ratio remained below 100 mmHg during the experimental protocol in both groups. This ratio achieved 52 ± 8 and 62 ± 4 mmHg in the LPV and TLV groups, evidencing similar severity of ARDS in both conditions.

**Fig. 2:**
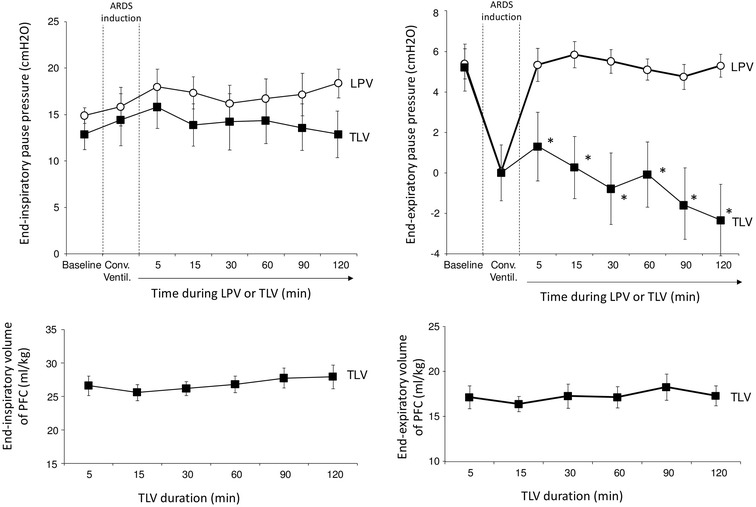
Airways pressures during end-inspiratory and end-expiratory pauses (upper panels) in rabbits presenting experimental acute respiratory distress (ARDS) and treated by gaseous lung protective ventilation (LPV) or total liquid ventilation (TLV), respectively. Lower panels illustrate end-inspiratory and end-expiratory volumes with perfluorocarbons (PFC) in the TLV group. *Conv. Ventil., non*-*protective conventional ventilation; n *= 8 *in each* LPV *and TLV group*; *p < 0.05 versus LPV

As shown in Fig. 2, end-inspiratory and expiratory airway pressures tended to decrease during TLV as compared to LPV. This difference was not statistically different for end-inspiratory pressures (*p* = 0.08) but only for end-expiratory pressures (5.3 ± 0.6 vs. − 2.4 ± 1.6 cmH_2_O at the end of the procedure, *p* < 0.001). Lung compliance was not significantly different between these two groups (Table 1). In the TLV group, lung liquid volumes averaged 25–30 and 15–20 ml/kg at the end of expiratory and inspiratory period (Fig. 2), respectively.

### Effect of hypothermic TLV on temperature, hemodynamic and bicarbonate blood levels

As shown in Table 1, body temperatures decreased very rapidly after TLV initiation as compared to LPV. This was associated with a decreased heart rate, as well as improved systemic arterial pressure. Mean arterial pressure achieved 74 ± 9 versus 56 ± 8 mmHg at the end of the procedure period in the TLV vs. LPV groups (*p* < 0.05), respectively. As shown in Table 2, the drop in bicarbonates arterial levels was also significantly attenuated by hypothermia in the TLV group as compared to LPV. Bicarbonates arterial levels achieved 31 ± 2 versus 24 ± 2 mmol/l in TLV versus LPV at the end of the protocol (*p* = 0.007).

### Effect of hypothermic TLV on inflammatory response assessed by broncho-alveolar lavage and lung histology

At the end of the protocol, the protein content of the broncho-alveolar lavage solution was similar between groups (351 ± 11 vs. 334 ± 13 µg/ml in TLV vs. LPV groups, respectively). Conversely, the concentration in interleukin-1β tended to decrease in TLV versus LPV, but the difference was not significant (7.1 ± 0.7 vs. 11.2 ± 2.5 pg/ml; *p* = 0.08).

**Fig. 3:**
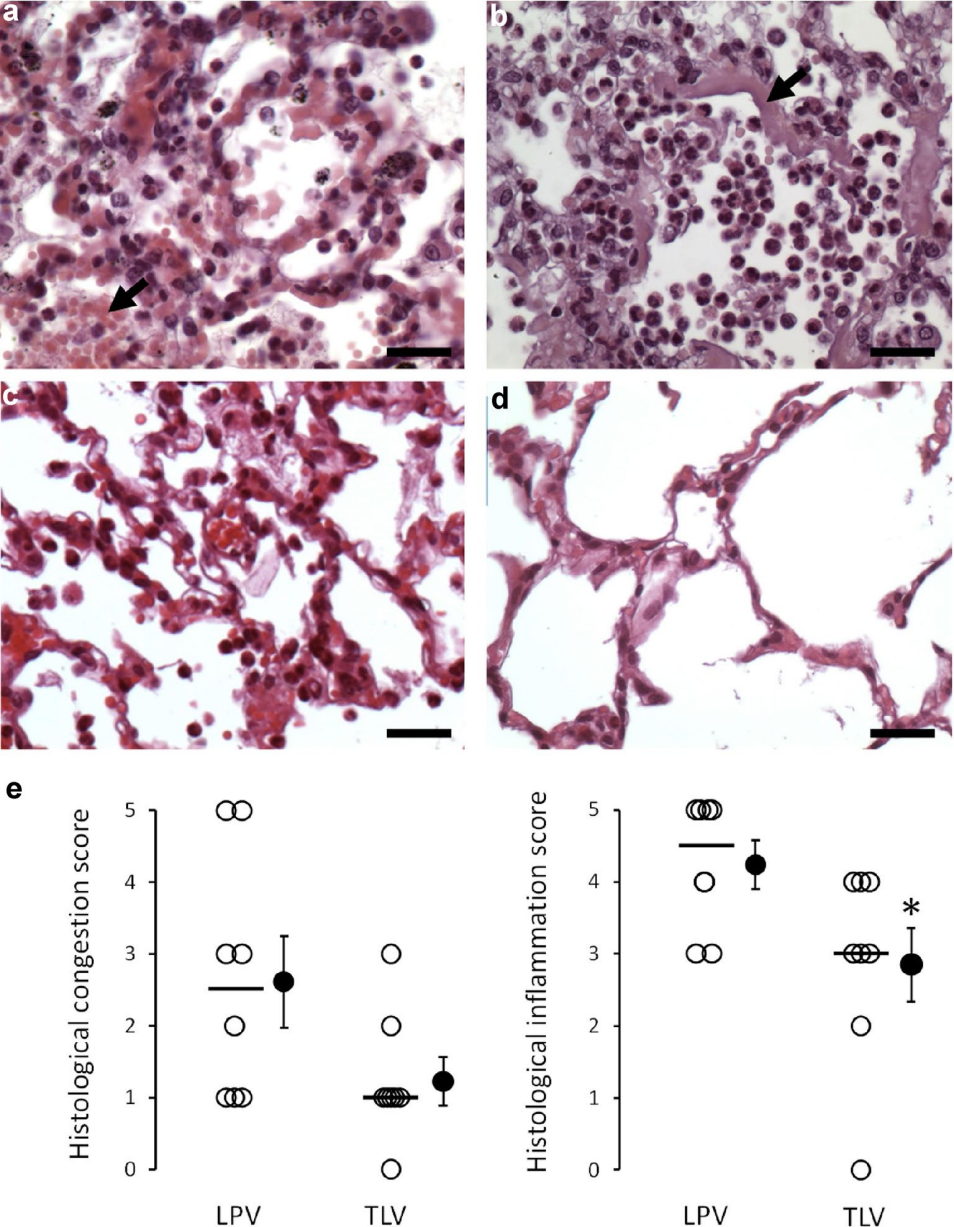
Histological appearance of the lungs in the group submitted to gaseous lung protective ventilation (LPV) or total liquid ventilation (TLV). ** a** Histological appearance of the lung in a rabbit from the LPV group with leukocytic alveolitis and severe congestion, evidenced by intra-alveolar red blood cells infiltration (arrow; Bar = 25 µm).** b** Severe lesions of leukocytic alveolitis in a rabbit from the LPV group. The arrow illustrates hyaline membranes, as a marker of severe alveolar lesions (Bar = 25 µm).** c** Histological appearance of the lung in a rabbit from the TLV group with moderate alveolitis and congestion (Bar = 25 µm).** d** Normal histological appearance of the lung in a rabbit from the TLV group (Bar = 25 µm).** e** Histological scores of the severity of the congestive and inflammatory lesions in the different groups (*n* = 8 in each LPV and TLV group). Open circles represent the individual value of each animal, and the bold line illustrates the median score of each group, respectively. Closed circles represent mean and standard of the mean of each group. *p < 0.05 versus LPV

After completion of the protocol, typical signs of ARDS were observed in all animals at lung histology. As illustrated in Fig. 3a, we observed moderate to severe leukocytic alveolitis and intra-alveolar red blood cells infiltration in all animals in the LPV group. Two animals also presented alveolar hyaline membranes, evidencing major ARDS lesions in this group. In the TLV group, lungs showed normal appearance in one animal and mild to severe inflammation and congestion in others. As shown in Fig. 3e, the blindly attributed score of inflammation was significantly reduced in TLV versus LPV group (median score = 3 vs. 4.5, respectively; *p* = 0.03). Conversely, the score attributed for congestion severity was not significantly different among groups (median score = 1 vs. 2.5; *p* = 0.08).

## Discussion

In the present study, hypothermic TLV did not modify gas exchange and lung compliance but attenuated hemodynamic failure and lung inflammation in a model of experimental aspiration-associated ARDS in rabbits. These demonstrations were obtained with a last-generation liquid ventilator which accurately controls lung volumes and pressures during TLV [13]. Importantly, the design of the study does not able to differentiate the effect of TLV from hypothermia since we investigated the effect of hypothermic TLV. Our ultimate goal is indeed to translate hypothermic TLV from the bench side to human practice for the treatment of the post-cardiac arrest syndrome [7–11].

Importantly, there are apparently conflicting results regarding the effect of liquid ventilation during ARDS in the literature. In 2006, Kacmarek et al. [22] demonstrated that partial liquid ventilation did not improve outcome as compared to conventional mechanical ventilation and recommended against its use in ARDS patients. In the latter study, partial liquid ventilation was performed after lung filling with 10 ml/kg or 20 ml/kg of perflubron, with high values of positive end-expiratory pressure (13 cmH2O) and relatively high tidal volumes for gas ventilation (≈ 8–10 ml/kg). In addition, patients were repeatedly derecruited by abrupt decreases in PEEP from 13 to 0 cmH_2_0, with putative air trapping [23]. Our strategy was completely different as we performed TLV rather than partial liquid ventilation, and we used the pressure and volume-control mode of the ventilator [16]. This led to much lower and likely safer airways pressures, averaging 15 cmH_2_O at end-inspiration, which is approximately 2 times lower than previously observed during partial liquid ventilation in ARDS patients [22]. In addition, hypothermic TLV is expected to be instituted for very short periods only to induce hypothermia after cardiac arrest, which makes it much easier to implement than prolonged liquid ventilation.

Beyond partial liquid ventilation, previous strategies of TLV were also tested in animal models of ARDS in the past. Most studies used much higher filling volume (20–30 ml/kg) and tidal volume (20–30 ml/kg) than we did in the present study [13, 17, 19]. For example, Pohlmann et al. improved gas exchanges in sheep after experimental ARDS using TLV with an initial filling with 30 ml/kg of perfluorocarbons, a frequency of 5 cycle per min and a tidal volume of 15–20 ml/kg during 24 h [17]. In another report, Avoine et al. used similar TLV parameters which improved gas exchanges after meconium aspiration [19]. However, such respiratory parameters during TLV were associated with high pulmonary pressures, e.g., 28–30 cmH_2_O for TLV peak pressures which might again be poorly tolerated on the longer term. In comparison, airways pressures were much lower in the present study. Lung volumes were also strongly reduced, i.e., below 15–20 ml/kg for expiratory residual volumes. Using such parameters, we were not able to improve gas exchanges as compared to LPV but a compromise should be made between the short-term need of gas exchanges improvement and the long-term hazards of lung trauma. In previous reports, we indeed demonstrated that low-volume TLV was much better tolerated regarding systemic and pulmonary hemodynamic [24]. In addition, hypothermic TLV could be done with low tidal volumes with no significant impact on gas exchanges, due to the reduced oxygen demand and carbon dioxide production during hypothermia. This is another argument in the favor of hypothermic TLV which is likely easier to implement in patients for these reasons. In the context of hypothermic TLV for post-cardiac arrest treatment, we could therefore speculate that such procedure is safe in both healthy [25] and damaged lungs after aspiration.

Interestingly, we also observed that hypothermic TLV could exert anti-inflammatory effects in the present report regarding lung histology. Such findings were already demonstrated with perfluorocarbons [26] or after experimental ARDS with other TLV devices. As example, Wolfson et al. [13] demonstrated that TLV could limit inflammation after oleic acid-induced ARDS. This could also be related to the lavage properties of TLV in a pulmonary aspiration setting. Indeed, Avoine et al. [19] demonstrated potent lavage properties of TLV in an animal model of meconial aspiration. This could open perspective for the treatment of pure ARDS, beyond the management of the post-cardiac arrest syndrome. For instance, a recent clinical study reported possible benefits with 48 h of cooling at 34–36 °C in 8 ARDS patients receiving neuromuscular blockade agents [27]. In neonates, a retrospective analysis also suggested consideration of therapeutic hypothermic as an adjunctive therapy during meconial aspiration syndrome [28].

Overall, the present study further supports that hypothermic TLV could be a supplementary tool in the armamentarium against post-cardiac arrest syndrome, acting on several facets of this dreadful syndrome. In previous studies, we demonstrated potent cardiac, neuro- and renal protection after experimental cardiac arrest after both shockable [8, 11, 29] and non-shockable rhythms [9]. More broadly, we reported strong attenuation of clinical and biological manifestations of multi-organ failure after aortic cross-clamping in a severe model of multi-organ ischemia reperfusion mimicking low perfusion states. This converges toward potent organ protection with hypothermic TLV after cardiac arrest, targeting the main component of the post-cardiac arrest syndrome. The current study further shows that hypothermic TLV might also be safely used after lung impairment and aspiration pneumonia, which carries specific morbidity after cardiac arrest through increased mechanical ventilation duration and ICU length of stay [1, 2].

Finally, this study presents several limitations. The main one is related to the experimental model which demonstrated very severe ARDS. As example, gas exchanges were dramatically altered, even after the initiation of LPV or TLV. This could explain the lack of actual benefits of TLV on gas exchanges. However, this further emphasizes that this strategy could be instituted safely even after lung injury. Importantly, the severity of ARDS in this model was previously described in rabbits submitted to HCl-induced lung injury [30]. In the latter study, PaO_2_/FiO_2_ ratio decreased below 100 mmHg after 105 min in control conditions with conventional mechanical ventilation [30]. Another limitation is that we did not induce cardiac arrest in this model. This could have been relevant since our ultimate goal is to implement TLV in the post-resuscitation setting. The protective effect of hypothermic TLV could have been different after such global ischemia–reperfusion injury. However, since we previously tested the effect of hypothermic TLV after cardiac arrest from respiratory cause [9], we chose to focus the present study on the pulmonary effect of TLV. Finally, we did not investigate the proper effect of normothermic TLV in the present study, which could be of interest to decipher the effect of TLV by itself on inflammation and hemodynamic status, independently from the rapid temperature management induced by TLV. The investigation of another group with “slow” cooling using other methods could also be of interest to strengthen our conclusions. All these comparisons deserve further investigations.

## Conclusion

In conclusions, we demonstrated that hypothermic TLV can be safely induced in rabbits during aspiration-associated ARDS. It does not improve gas exchange and lung compliance but improves hemodynamic parameters and attenuates lung inflammation. This deserves further investigation after cardiac arrest-associated ARDS.

## Abbreviations

ARDS: acute respiratory distress syndrome
PaO_2_: partial pressure of oxygen
PaCO_2_: partial pressure of carbon dioxide
PEEP: positive end-expiratory pressure
LPV: lung protective ventilation
PFO: perfluoro-n-octane
TLV: total liquid ventilation
